# NMFBFS: A NMF-Based Feature Selection Method in Identifying Pivotal Clinical Symptoms of Hepatocellular Carcinoma

**DOI:** 10.1155/2015/846942

**Published:** 2015-10-12

**Authors:** Zhiwei Ji, Guanmin Meng, Deshuang Huang, Xiaoqiang Yue, Bing Wang

**Affiliations:** ^1^Machine Learning & Systems Biology Lab, School of Electronics and Information Engineering, Tongji University, 4800 Caoan Road, Shanghai 201804, China; ^2^School of Information Engineering, Zhejiang A&F University, 88 Huancheng North Road, Linan 311300, China; ^3^Department of Clinical Laboratory, Tongde Hospital of Zhejiang Province, 234th Gucui Road, Hangzhou 310012, China; ^4^Department of Traditional Chinese Medicine, Changzheng Hospital, Second Military Medical University, 415 Fengyang Road, Shanghai 200003, China; ^5^The Advanced Research Institute of Intelligent Sensing Network, Tongji University, 4800 Caoan Road, Shanghai 201804, China; ^6^The Key Laboratory of Embedded System and Service Computing, Tongji University, 4800 Caoan Road, Shanghai 201804, China

## Abstract

*Background*. Hepatocellular carcinoma (HCC) is a highly aggressive malignancy. Traditional Chinese Medicine (TCM), with the characteristics of syndrome differentiation, plays an important role in the comprehensive treatment of HCC. This study aims to develop a nonnegative matrix factorization- (NMF-) based feature selection approach (NMFBFS) to identify potential clinical symptoms for HCC patient stratification. *Methods*. The NMFBFS approach consisted of three major steps. Firstly, statistics-based preliminary feature screening was designed to detect and remove irrelevant symptoms. Secondly, NMF was employed to infer redundant symptoms. Based on NMF-derived basis matrix, we defined a novel *similarity measurement of intersymptoms*. Finally, we converted each group of redundant symptoms to a new single feature so that the dimension was further reduced. *Results*. Based on a clinical dataset consisting of 407 patient samples of HCC with 57 symptoms, NMFBFS approach detected 8 irrelevant symptoms and then identified 16 redundant symptoms within 6 groups. Finally, an optimal feature subset with 39 clinical features was generated after compressing the redundant symptoms by groups. The validation of classification performance shows that these 39 features obviously improve the prediction accuracy of HCC patients. *Conclusions*. Compared with other methods, NMFBFS has obvious advantages in identifying important clinical features of HCC.

## 1. Introduction

Hepatocellular carcinoma (HCC) is the third most common cause of cancer-related death worldwide and the leading cause of death in patients with the complication of cirrhosis [1, 2]. The occurrence of HCC is larvaceous and short of specific symptoms [3, 4]. Its diagnosis depends on biopsy, imaging examination such as Doppler ultrasound, computed tomography, magnetic resonance imaging, and blood test [5, 6]. Once the patients with HCC see doctors, the disease has often entered its late stage, losing the chance of resection. Hence, seeking simple methods to predict HCC and its clinical stage is very meaningful and helpful to improve the diagnosis of HCC.

As one of the most popular complementary and alternative medicine modalities, Traditional Chinese Medicine (TCM) plays an active role in treatment of malignant tumors including HCC in Chinese and some East Asian countries [7, 8]. Unlike modern medicine, the diagnosis and treatment of TCM depend on the analysis of symptoms and signs of HCC collected by inspection, auscultation and olfaction, inquiry, and pulse taking and palpation [8]. TCM regards specific combination of symptoms and signs as a TCM syndrome, which is the main basis for treatment; and it can be also used to guide clinical diagnosis of HCC. Our previous work proposed a hierarchical feature selection (PSOHFS) model to quickly identify the potential HCC syndromes from a TCM clinical dataset [9], by which all the original symptoms were classified into several groups according to the categories of clinical observations, and each symptom group was then converted into a syndrome signature to reduce the searching space of feature selection. But the limitation of this method is that the interactions among symptoms which belong to different categories (aspects) were ignored. Therefore, the current challenge is to design an efficient feature selection approach for high-dimensional TCM data with consideration of clinical significance.

In this study, a nonnegative matrix factorization- (NMF- [10]) based feature selection (NMFBFS) method was proposed to select pivotal clinical symptoms for HCC diagnoses. A TCM clinical dataset was used in this work, which consisted of 407 HCC patients with 57 clinical symptoms. Each patient sample is labeled with a clinical-staging symbol which indicates the severity of certain patient. Firstly, the preliminary screening with statistical method was designed to detect irrelevant symptoms from the full symptom set. Secondly, the process of NMF was implemented after eliminating the irrelevant symptoms. Based on the NMF-derived basis matrix, we defined a similarity measure to infer redundant symptoms by calculating the distance and correlation among the symptoms. Finally, the secondary dimension reduction was implemented based on the inferred groups of redundant symptoms. We converted each symptom group to a new feature (named “mixed feature”) if these symptoms represent similar distribution patterns on the sample space. The experiment results show that 39 novel features inferred by NMFBFS obviously improve the accuracy of diagnosis of HCC clinical samples. Moreover, NMFBFS-derived 39 optimal clinical features included some well-known common symptoms of HCC patients. Comparing to three representative feature selection methods (ReliefF [11], mRMR [12], and Elastic Net [13]), our proposed approach showed the best performance to identify optimal clinical features for HCC patients.

## 2. Materials and Methods

### 2.1. Experimental Data

#### 2.1.1. Description

In this work, the questionnaire survey dataset of HCC includes 407 samples within two years, and each patient was observed on 57 clinical symptoms (Table 1). Each patient sample is labeled with a symbol of clinical stage, which is related to TCM pattern of syndrome and indicates the severity degree of HCC. According to the international staging system [14], there are three stages and two substages in each phase in this dataset. The aim of our work is to identify the symptom signatures, which are related to three clinical stages: phases I, II, and III, and the larger value indicates that stronger positive symptom occurred. Within our dataset, all the original symptoms are described by two types of data: binary (0 or 1) or integer (0, 1, 2, 3,…). For example, the type of symptom “tinnitus” is binary (0 or 1), which means two possible states: occurrence (positive) or nonoccurrence (nonpositive). Another example is “sleeplessness” whose value can be 0, 1, 2, or 3. The larger the value is, the stronger the positive state will be. A symptom does not appear positive if its value equals zero.

#### 2.1.2. Data Preprocessing

*Refinement of Feature Set*. Our original dataset consists of 407 HCC patient samples (Table 1). The first step of preprocessing is to remove the useless features because they provide no useful information for the following classification. If a feature is constant on all the observed samples, it can be considered as useless feature. For our dataset, some symptoms, such as “pale tongue” and “slow pulse,” were removed out because there is no any observed patient positive on these symptoms. After removing this kind of features, a refined clinical dataset with 407 samples and 57 symptoms (*V*_1_,…, *V*_57_) can be obtained.

*Simplification of Clinical Staging*. The clinical staging of HCC patients in our original dataset was marked with collections “IA,” “IB,” “IIA,” “IIB,” “IIIA,” and “IIIB.” For identifying the symptom signatures related to three clinical stages, all the samples would be relabeled as three classes. Here, we remarked class label “1” for the samples labeled “IA” and “IB.” In a similar way, class label “2” is used for “IIA” and “IIB” and “3” is for “IIIA” and “IIIB.” Finally, all the 407 clinical samples can be distributed in three categories: 82 samples in phase I, 195 in phase II, and 130 in phase III. The details of the refined dataset were described in Table 1.

### 2.2. Feature Selection

Feature selection can be organized into three categories, depending on how they interact with the construction of model. Filter methods employ a criterion to evaluate each feature individually and are independent of the model [15]. Among them, feature ranking is a common method which involves ranking all the features based on a certain measurement and selecting a feature subset which contains high ranked features [16]. However, one of the drawbacks of ranking methods is that the selected subset might not be optimal in that a redundant subset might be obtained. Wrapper methods involve combination searches through the feature space, guided by the predicting performance of a model [17]. Heuristic search is widely used in wrapper methods as searching strategy which can produce good results and is computationally feasible; however, they often yield local optimum results. For an embedded method, the feature search process is embedded into classification algorithm, so that the learning process and the feature selection process cannot be separated [18].

### 2.3. Nonnegative Matrix Factorization

Nonnegative matrix factorization (NMF) aims to obtain a linear representation of multivariate data under nonnegativity constraints. These constraints lead to a part-based representation because only additive, not subtractive, combinations of the original data are allowed [19]. In general, NMF can be used to describe hundreds to thousands of features in a dataset in terms of a small number of metafeatures, particularly in gene expression profiles analysis [20–22].

Let *X* be *n* × *p* nonnegative matrix; that is, each element *x*_*ij*_ ≥ 0 in *X*. Nonnegative matrix factorization (NMF) consists in finding an approximation

(1) $$\begin{array}{c} X\approx WH, \end{array}$$

where the* basis matrix W* and the* mixture coefficient matrix H* are *n* × *r* and *r* × *p* nonnegative matrices, respectively, where *r* > 0 and *r* ≪ min⁡(*n*, *p*). The objective behind the small value of *r* is to summarize and split the information contained in *X* into *r* factors (also called “basis” or “metafeature”). The matrix *H* has the same number of samples but much smaller number of features rather than matrix *X*. Therefore, the metafeature expression patterns in *H* usually provide a robust clustering of samples [22].

The main approach to NMF is for solving estimate matrices *W* and *H* as a local minimum:

(2) [D(X,WH)+R(W,H)]W,H≥0min⁡,

where *D* is a loss function that measures the quality of the approximation which is usually based on either the Frobenius distance or the Kullback-Leibler divergence [19]. *R* is an optional regularization function, defined to enforce desirable properties on matrices *W* and *H*, such as smoothness or sparse [23, 24].

In our study, the loss function in NMF is based on Kullback-Leibler divergence [25]. The above function *R* was defined as follows:

(3) $$\begin{array}{c} R\left(\;W,H\;\right)=F_{1}\left(\;W\;\right)+F_{2}\left(\;H\;\right), \end{array}$$

where *F*_1_(*W*) and *F*_2_(*H*) are regulation functions for *W* and *H*, respectively. Here, we applied Tikhonov smoothness regularization [26] for *W* in

(4) $$\begin{array}{c} F_{1}\left(\;W\;\right)=\frac{1}{2}\underset{i,j}{\sum }{\left(\;{\left[\;W\;\right]}_{ij}-c\;\right)}^{2}, \end{array}$$

where *c* is a constant positive or zero. In addition, we applied sparsity-enforcing regularization [26] for *H* in

(5) $$\begin{array}{c} F_{2}\left(\;H\;\right)=\frac{1}{2}\underset{j}{\sum }{\left(\;{\left\|\;{\left[\;H\;\right]}_{.j}\;\right\|}_{2}^{2}-\alpha^{2}{\left\|\;{\left[\;H\;\right]}_{.j}\;\right\|}_{1}^{2}\;\right)}^{2}. \end{array}$$

In formula (5), [*H*]_.*j*_ is *j*th row of *H*. ‖[*H*]_.*j*_‖_2_^2^ and ‖[*H*]_.*j*_‖_1_^2^ define the *l*_2_-norm and *l*_1_-norm of [*H*]_.*j*_. The algorithm proposed by Lee is a well-established method to solve the optimization of NMF [27].

### 2.4. NMF-Based Feature Selection

**Figure 1:**
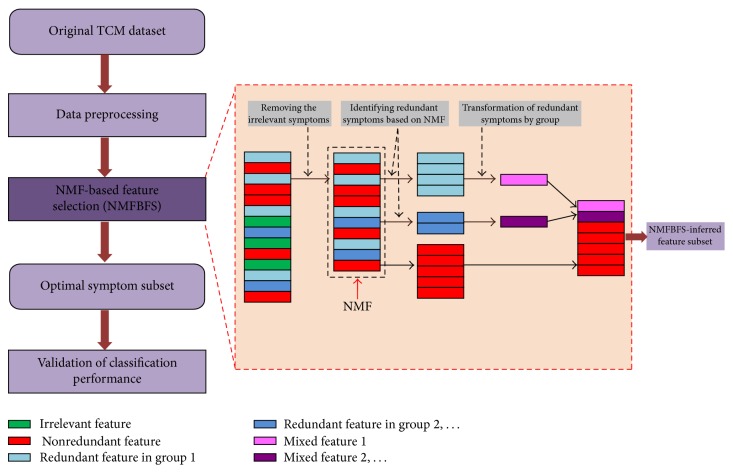
The flowchart of the proposed approach.

In this study, our proposed NMF-based feature selection (NMFBFS) approach can be seen as a two-stage filter method. In the first stage, preliminary screening is implemented to detect irrelevant symptoms and remove them from the whole feature set. In the second stage, NMF clusters the redundant symptoms which potentially have similar patterns into different groups, and each group is then transformed into new single features to reduce the dimension. Obviously, the process of NMFBFS is independent of classifier and can quickly infer the optimal feature subset even in the high-dimensional dataset. The flowchart of NMFBFS is shown in Figure 1.

#### 2.4.1. Removing the Irrelevant Symptoms

In our questionnaire, all the symptoms were defined by clinical doctors, which covered many aspects of patients. However, the relevance weight of each feature for distinguishing samples among the clinical stages was not quantitatively studied. In machine learning, the irrelevant features provide no useful information in any context and always scarcely contribute to patient stratification [28]. If the sample size is large, it is meaningful to quickly detect the irrelevant symptoms by calculating the frequencies of positive. Here, we calculated the ratio (frequency) of presence (positive) of each symptom on the samples in every clinical stage. If the frequencies of certain symptom in all the clinical stages are very low, which indicates that this symptom hardly appears positive in most of patients, therefore it is considered as an irrelevant symptom. After removing the irrelevant symptoms from the original dataset, the rest of symptoms are considered as relevant features, which are potentially related to at least one class of patients (or one clinical stage).

#### 2.4.2. Identifying Redundant Symptoms Based on NMF

After the irrelevant symptoms had been removed, nonnegative matrix factorization was applied on the dataset *X* (*n* × *p*). For a given rank *r*, the matrix *X* can be decomposed to* basis matrix W* and* coefficient matrix H*. Usually, the value of rank *r* is much smaller than the number of features (*n*) and the sample number (*p*), so that there is at least one dimension in both *W* and *H* being very small. The widespread appliances of NMF in biclustering further indicate that basis matrix *W* can be used for feature clustering and coefficient matrix *H* is used for sample clustering, respectively [20, 21]. In our study, the number of samples is much larger than the dimensionality; hence, directly calculating distance or correlation to measure the similarity between original features (symptoms) on all the samples will lead to biases because some features might represent local similar patterns on a part of samples. Fortunately, the basis matrix *W* represents the compressed sample space of matrix *X*, which facilitates uncovering the difference between features. Here, we introduced two features (*v*_*i*_ and *v*_*j*_) in original dataset *X* as an example to clarify the basic idea of this step. According to the definition of NMF, we can easily know

(6) xi=wi×H,xj=wj×H,

where *x*_*i*_ and *x*_*j*_ are *i*th and *j*th rows of matrix *X*; *w*_*i*_ and *w*_*j*_ are *i*th and *j*th rows of matrix *W*. The following can be easily found. (1) If *w*_*i*_ ≈ *w*_*j*_, then *x*_*i*_ ≈ *x*_*j*_; (2) if *w*_*i*_ = *kw*_*j*_, then *x*_*i*_ = *kx*_*j*_, where *k* is a constant. Furthermore, if *i*th row *w*_*i*_ in matrix *W* is very close to *w*_*j*_, the feature *v*_*i*_ might have a similar pattern as *v*_*j*_ on all the samples. Therefore, we defined a novel* similarity measurement* in formula (7) to approximately evaluate redundancy between the two original symptoms via matrix *W*:

(7) simvi,vjsimwi,wj=sim_distwi,wj+sim_corrwi,wj2,

where

(8) sim_distwi,wj=1−wi−wj×wi−wjTMax⁡D,

(9) sim_corrwi,wj=wi−w−×wj−w−Twi−w−×wi−w−T×wj−w−×wj−w−T.

Formula (8) uses* distance-based similarity*, which indicates how two corresponding features are close to each other; and formula (9) adopts* correlation-based similarity*, which describes similar patterns of two original features. Hence, our developed similarity measurement considered distance and correlation between features at the same time. Max⁡*D* in formula (8) is the maximal distance value in all pairs of (*w*_*i*_, *w*_*j*_). Based on the above definition of similarity, we further calculated the similarity matrix SMX using all the basis rows in *W* (SMX(*i*, *j*) = sim(*v*_*i*_, *v*_*j*_)), where element SMX(*i*, *j*) denotes the similarity between original features *i* and *j*. Given a threshold *θ* (0 < *θ* < 1), we can screen all the redundant features by groups with SMX(*i*, *j*) > *θ*.

#### 2.4.3. Transformation of Redundant Symptoms by Group

In the above section, all the redundant symptoms were screened out and were organized into different groups. For each symptom group, a new mixed feature was extracted as the representation of the whole group and replaced all the original features within this group. Therefore, NMFBFS-inferred optimal feature subset includes two parts: nonredundant original features and new generated mixed features (see Figure 1). There are two strategies that can be used to transform the redundant symptom groups to mixed features.

(1) Calculate the mean vector from all the redundant symptoms as in

(10) $$\begin{array}{c} x_{NF}=\text{mean}\left(\;x_{r1},x_{r2},\ldots ,x_{rn}\;\right), \end{array}$$

where *x*_*r*1_, *x*_*r*2_,…, and *x*_*rn*_ are the feature vectors of original dataset *X* and are determined as redundant symptoms in a group. *n* denotes the number of inferred redundant symptoms in a group. The vector *x*_*NF*_ of new single feature *v*_*NF*_ was averaged on that group.

(2) Randomly select a vector from one of redundant symptoms as

(11) $$\begin{array}{c} x_{NF}\in \left\{\;x_{r1},x_{r2},\ldots ,x_{rn}\;\right\}. \end{array}$$

In our study, we transformed the groups of redundant symptoms to new mixed features by using formula (10). After this step, the feature space of the clinical dataset was further reduced so that the optimal feature subset rarely included redundant features.

## 3. Simulation Design

Firstly, we calculated the frequencies of each original symptom appearing positive at each clinical stage and then removed the irrelevant symptoms if their frequency values were very low.

Secondly, a representative sample set was screened out for NMF analysis. In our dataset, the number of samples in three phases of HCC varies a lot, that is, from 82, 130 to 195. If the whole dataset is used, a class imbalance problem will be caused [29–31]. In addition, the sex ratio of patients is also seriously unbalanced in the original dataset (Table 1). For avoiding the bias caused by imbalance of samples, we selected 40 samples from each clinical phase with equal proportion of male and female (20 : 20) to construct a representative clinical dataset *D*_*R*_ (120 samples in total) for the following NMF analysis. Considering the fact that each original sample has a class label which corresponds to clinical stage of that patient, for all the original samples (407), we can actually get a preliminary participation of samples as three clusters, which can also be considered as a trained KNN clustering model [32]. We then defined the center of each cluster, which is the mean vector of all the samples in the same cluster. Given a large value of *K*, we input each center of cluster into the above KNN model and keep the output consistent with the corresponding class label of the center. Based on the *K*-nearest neighbors, we can finally screen out 40 representative samples (20 males and 20 females) of each clinical stage according to Euclidean distance.

Finally, several redundant symptom groups were identified. Then we transformed each redundant symptom group into a new mixed feature. Combining all the nonredundant original features with new generated mixed features, we obtained an optimal clinical symptom subset of HCC. At last, the classification performance of this feature subset was further validated by least squares support vector machines (LSSVM) [33, 34].

*Experimental Parameters*. At first, we set a frequency threshold to identify the irrelevant symptoms. The NMF *R* package [35] was then employed as a computational framework for nonnegative matrix factorization algorithms in *R*. For this method, the optimal rank *r* should be determined firstly. Currently there are several approaches that had been proposed to determine the optimal value of *r* [36, 37]. In our study, two methods, that is, cophenetic coefficient [36] and RSS curve [37], had been adopted to determine the optimal rank *r* range from 2 to 7. After obtaining the results of NMF with optimal *r*, we calculated the similarity matrix SMX with all the basis rows and inferred the redundant symptoms with a threshold *θ* = 0.95, which meet the following conditions: sim_corr(*w*_*i*_, *w*_*j*_) ≥ 0.95 and sim_dist(*w*_*i*_, *w*_*j*_) ≥ 0.95 in formulas (7)–(9). Finally, a LSSVM classifier had been implemented to validate the classification performance of inferred optimal symptom subset. In the LSSVM multiclass model, Gaussian RBF kernel was employed, and the kernel parameters *σ* ^2^ and *γ* were determined by grid search [38]. In our grid search, we set *σ* ^2^ = 10^*a*^ and *γ* = 10^*b*^. Variable *a* changes from −1 to 5 with step 0.25, and variable *b* changes from −1 to 4 with step 0.2. Therefore, we have the range of [0.1,100000] for *σ* ^2^ and the range of [0.1,10000] for *γ*. Totally, there are 24 levels for the value of *σ* ^2^ and 25 levels for *γ*. In other words, there are 600 pairs of *σ* ^2^, *γ* tested when training a LSSVM classifier. To find an optimal value of *σ* ^2^, *γ*, we used 5-fold cross-validation to evaluate the classification accuracy of LSSVM model.

## 4. Results and Discussion

Firstly, we calculated the frequencies of positive for all the original symptoms (57) at each clinical stage (see Supplementary Table S1 available online at http://dx.doi.org/10.1155/2015/846942). Eight irrelevant symptoms were judged as irrelevant features (threshold: 10%). From Table 2, we can clearly see that these symptoms appeared on few patients (less than 10% in each clinical stage) in the clinical observation and therefore they were considered as noisy features in the process of diagnosis. Because the total number of samples is large (407), we considered that the eight irrelevant symptoms identified with statistical analysis are very reliable. A part of symptoms shown in Table 2 was proved by previous studies. For example, Lai et al. concluded that no association is detected between “emotional depression” and the risk of hepatocellular carcinoma in older people in Taiwan [39, 40]. In addition, Peng et al. studied 169 Chinese patients with HCC; only three patients presented with hydrothorax, which also indicated that this symptom was not a key symptom in the process of liver cancer development [41, 42]. In addition, “edema in lower extremities” is undoubtedly a well-known symptom of HCC patients in clinic [43]; however, it was considered an irrelevant symptom in this study because it rarely appeared in all the three stages of our data. Increasing the observed samples or reducing the threshold will make it as a candidate symptom.

**Figure 2:**
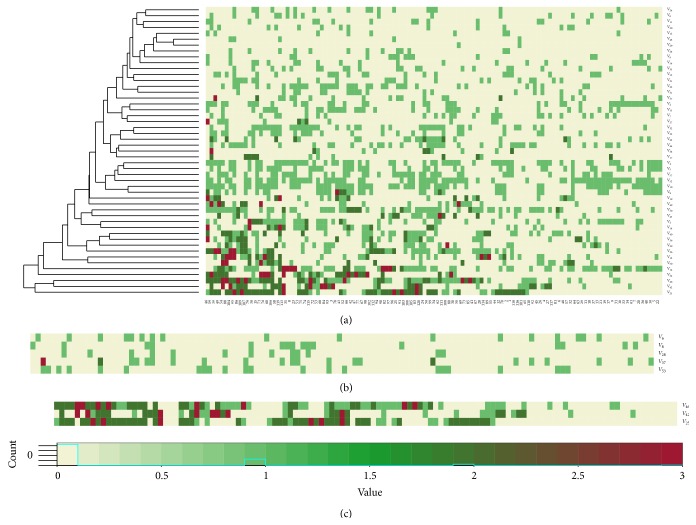
The heatmap of the representative clinical dataset *D*_*R*_. (a) The heatmap of *D*_*R*_ with 49 symptoms and 120 samples. (b) The distribution patterns of symptoms *V*_6_, *V*_8_, *V*_28_, *V*_37_, and *V*_53_ indicate that the frequencies of positive are low. (c) The distribution patterns of symptoms *V*_46_, *V*_42_, and *V*_25_ indicate that the frequencies of positive are high.

**Figure 3:**
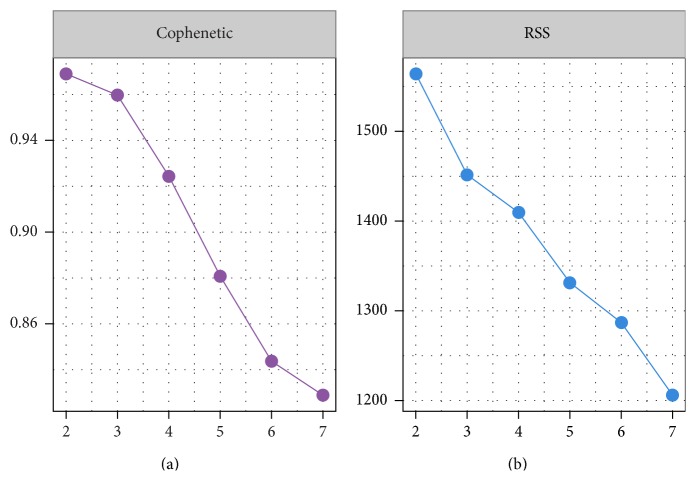
Estimation of the optimal rank *r*.

Secondly, the calculation of NMF was implemented after removing all the detected irrelevant symptoms. According to the description in “Simulation Design”, NMF was applied on the representative matrix *D*_*R*_ with 120 HCC samples, which uniformly covered three clinical phases.  Figure 2(a) represents the fact that *D*_*R*_ is a sparse matrix, in which large partition of elements is zero (no positive), such as symptom *V*_6_ shown in Figure 2(b). However, there are also some symptoms that were positive on many patients, such as symptom *V*_25_ shown in Figure 2(c). Matrix *D*_*R*_ does not show obvious subtypes and patterns; hence, it is hard to compare the similarity directly between symptoms with the row vectors of *D*_*R*_ since the number of samples is still very large. In this study, we used NMF to compress the representative matrix *D*_*R*_ and to reveal the distribution patterns of features (symptoms) on fewer samples. Before the calculation of NMF, a critical parameter should be firstly determined: the value of factorization rank *r*. According to Brunet's method, the first value of *r* for which the cophenetic coefficient starts decreasing is the optimal one [36]. Frigyesi and Höglund suggested choosing the first value where the RSS curve presents an inflection point [37]. Based on these two methods, we determined that “3” is a reasonable value of rank *r* for the clinical data matrix *D*_*R*_. The curves shown in Figure 3 also confirm this conclusion. Nonnegative matrix factorization was then implemented on the matrix *D*_*R*_ (49 × 120) with rank 3. It also indicates that the number of metafeatures (basis) equals 3.

**Figure 4:**
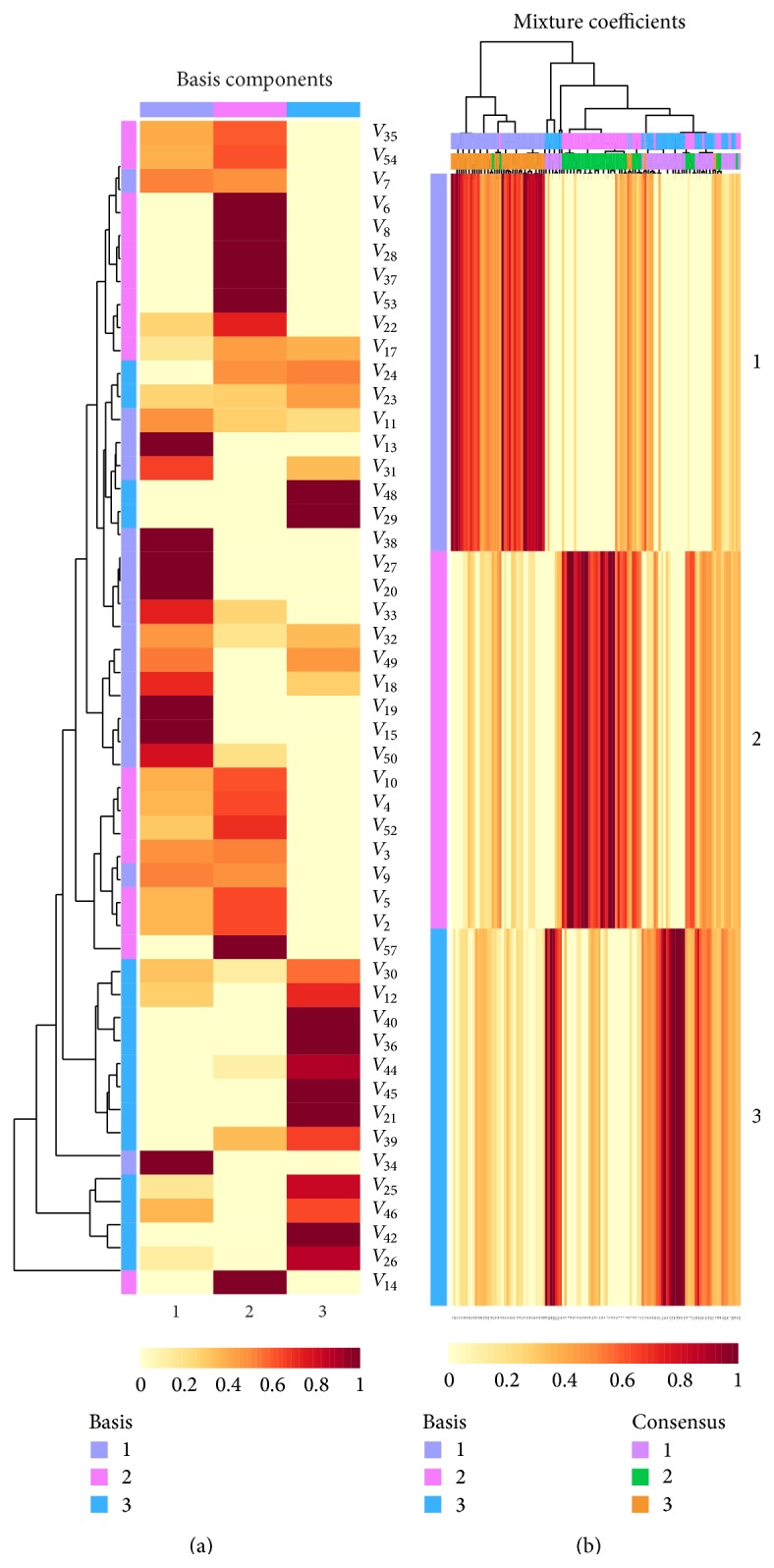
The result of NMF on the dataset *D*_*R*_. The left side indicates the visualization of matrix *W* (49*∗*3), and right side denotes matrix *H* (3*∗*120).

Figure 4 represents the final results of NMF which included the basis matrix *W* (49 × 3) and mixture coefficient *H* (3 × 120). Each row in matrix *W* uses a compressed pattern to approximatively represent the distribution of a symptom on all the original samples. Comparing with matrix *D*_*R*_ shown in Figure 2, the obvious difference in matrix *W* is that there are several groups of features revealing similar patterns in the compressed sample space, such as *V*_40_ and *V*_36_ in Figure 4. According to Figure 2(a), we can find that the distance between the vectors of symptoms *V*_40_ and *V*_36_ in *D*_*R*_ is also close; furthermore, the compressed patterns of *V*_40_ and *V*_36_ in matrix *W* (*w*_40_ and *w*_36_) in Figure 4 facilitate easier identifying of redundant features which have very similar distribution patterns.

**Figure 5:**
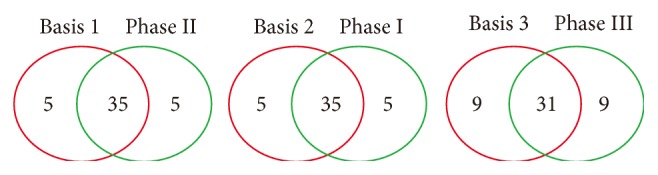
The relationships between NMF-derived basis components and clinical stages of samples.

The matrix *H* has the same number of samples but much smaller number of metafeatures (basis) rather than original matrix *X* [36]. Therefore, the metafeature expression patterns in *H* usually provide a robust clustering of samples. Given the *j*th column in *H* as *H*_*j*_ = [*h*_*j*1_, *h*_*j*2_, *h*_*j*3_]^*T*^, we determined that *j*th clinical sample is placed into *k*th cluster if max⁡(*H*_*j*_) = *H*_*j*_(*k*), where *k* ∈ {1,2, 3}. Hence, we used matrix *H* to group all the samples into 3 clusters, which correspond to 3 bases (metafeature).  Figure 5 shows that there are great overlaps between the clinical-staging markers (a priori knowledge of class labels) and indexes of basis components (metafeatures) on the 120 original clinical samples included in dataset *D*_*R*_.

In matrix *W*, each column also corresponds to a metafeature or basis (see Figure 4). Entry *w*_*ij*_ in matrix *W* is the coefficient of original feature *i* in metafeature (basis) *j* [36]. Therefore, an original feature *i* relates to certain basis *j* if *w*_*ij*_ is the largest entry in row *i* of matrix *W*. From Figure 4, we can clearly see that the original symptom features participating in the same basis have similar expression patterns rather than that in other bases.  Table 3 represents the symptoms which are related to all basis components. Combination of Figure 5 and Table 3 further indicates that the “basis 1” related symptoms are very related to the clinical samples of phase II, and “basis 2” and “basis 3” related symptoms are very related for phase I and phase III, respectively. This finding contributes to identifying* clinical phase-specific* important symptoms via NMF. Moreover, the partition of 49 clinical symptoms shown in Table 3 was well supported by some related studies. For example,* nausea* is observed as a common adverse effect in HCC patients in phase I [44]. The symptoms* ascites, anorexia, fever*, and* jaundice* often occurred in phase II [43, 45–48]. The symptoms “*yellow complexion*” and “*yellow skin and eye*” shown in Table 3 are obvious appearances of* jaundice*. For phase III,* pain* is the most obvious characteristic in HCC patients [49]. There are three pain-related symptoms presented in Table 3: “*pain in shoulder and back,*” “*chest pain,*” and “*distending pain in hypochondrium.*” Moreover,* fatigue* and* weakness* were also common in HCC patients [43]. Together, these findings suggest that NMF with an optimal rank can reveal the latent associations between the potential symptom features and clinical phases.

Just as mentioned above in “Simulation Design,” several groups of redundant features were then screened out according to a given threshold *θ* = 0.95 (Table 4). We obtained two redundant symptom groups from each basis component, which indicates that the redundant symptoms included in the same group also might have similar patterns in the original sample space. Here, we take Figures 2(b)-2(c) as examples to collaborate the effectiveness of our method. Figure 2(b) represents the distribution of positive of five symptoms in the dataset *D*_*R*_. These five symptoms (*V*_6_, *V*_8_, *V*_28_, *V*_37_, and *V*_53_) were identified as basis 2 related features, and they are most possibly belonging to phase I (Table 4). Although each of the row vectors in Figure 2(b) is not completely equal, they all represent relative lower frequency of positive (15.17 ± 3.25%) and their local distribution patterns are similar in a way. Comparing the corresponding rows of these five symptoms in matrix *W* in Figure 4, we found that the compressed patterns of these symptoms are very similar. Similarly, the symptoms (*V*_46_, *V*_42_, and *V*_25_) are potentially related to basis 3, the frequency of positive for each is over 50%, and the mean value of positive for these three symptoms is 1.77, which further indicate that they might be related to some patients whose conditions are very serious. Although the symptoms *V*_46_, *V*_42_, and *V*_25_ were not identified as redundant symptoms with given threshold (0.95), their compressed patterns in matrix *W* in Figure 4 also suggested that their patterns were very close. In summary, we considered a fact that the matrix *W* facilitates evaluating the difference among symptoms, and matrix *H* can validate the high degree of correlation between class labels of samples and basis indexes. After inferring the redundant symptoms with given threshold, we combined each symptoms' group together and converted it into a new feature (named mixed feature). Finally, we obtained 39 clinical features (FS_1_) of HCC as the optimal feature subset, which consisted of two parts: 33 original symptom features (FS_2_) and 6 new mixed features (FS_3_) (Table 5). Based on the analysis of results of NMF, the feature space of original dataset was further reduced.

For evaluating the potential of NMFBFS-inferred optimal feature subset, we firstly tested the classification accuracy of three candidate feature subsets FS_1_, FS_2_, and OFS on the training set (120 representative samples). FS_1_ and FS_2_ were generated by feature selection with the threshold *θ* (0.95). OFS denoted 49 original symptom features in the dataset *D*_*R*_.  Table 6 indicates that the 39 optimal features, which covered 33 original symptom features and 6 new mixed features, result in the best classification accuracy on the training samples. The performance of FS_2_ was much better than OFS; however, it was still worse than FS_1_ because the new mixed features also have important contributions to classification.

We then compared the performance of our NMFBFS with three well-known feature selection methods (ReliefF [11], mRMR [12], and Elastic Net [13]). ReliefF was implemented using MATLAB function. “mRMRe” and “elasticnet” *R* packages were applied for mRMR and Elastic Net based feature selection, respectively. Supplementary Figure S1 represents the ReliefF-based feature ranking. Supplementary Figure S2 represents the Elastic Net (*λ* = 0.5) solution paths for feature selection. We selected Top 20 features and Top 40 features as two candidate feature subsets for each method to evaluate their classification performances: FS_RF20_ and FS_RF40_ generated from ReliefF; FS_MR20_ and FS_MR40_ inferred from mRMR; FS_EN20_ and FS_EN40_ inferred from Elastic Net.  Table 7 represents the classification performance of the above six candidate feature subsets and the NMFBFS-derived optimal feature subset FS_1_ on the training set (120 representative samples). The results indicate that NMFBFS-inferred feature subset has the best classification accuracy in training samples.

Except 120 representative training samples which were screened out to implement the NMF analysis, the remaining samples can be used to test the classification accuracy of optimal feature subset. We randomly selected 40 samples (10 : 20 : 10 for each clinical stage) from the rest of the samples and then evaluated the classification accuracy of inferred feature subset by each method (NMFBFS, ReliefF, mRMR, and Elastic Net).  Table 8 shows the differences among all these methods, and it can be found that the optimal feature subset inferred by our proposed method has the best generalization performance.

Finally, the more important thing is that the selection of threshold *θ* determines how many groups of redundant symptoms will be screened out. Here, we further discussed the effects of threshold *θ* to the optimal feature subsets on the classification performance.  Table 9 shows the differences among three optimal feature subsets inferred by the proposed approach with different values for threshold *θ*. From Table 9, we can obviously see that the bigger value of *θ* will screen redundant symptoms strictly, which leads to less similar symptoms that would be obtained. With a smaller value of *θ*, much more symptoms can be categorized into the same groups; hence, the original feature space will be sharply reduced by our approach.  Table 9 denotes that, with the decrease of *θ*, the size of optimal feature subset was narrowed down but the classification accuracy was also decreased. These results suggest that a bigger value of *θ* will result in less redundant symptoms and therefore induce a larger size of optimal feature subset; oppositely, smaller *θ* can provide more redundant symptoms and sharply reduce the feature dimension. An extreme case is that *θ* equals “0,” which means that we can get one mixed feature for each basis and the size of optimal feature subset is equal to the number of bases. In a word, how to determine the value of *θ* depends on the size of optimal feature subset and its corresponding classification performance.

## 5. Conclusions

In this study, we developed the NMFBFS approach to efficiently extract the important clinical symptoms of HCC from clinical observation data. NMFBFS is a two-stage filter method for feature selection as follows. (1) In the first stage, preliminary screening is implemented to detect and remove the irrelevant features; (2) in the second stage, NMF was applied to identify the redundant features by groups which might represent similar distribution patterns. Each redundant symptom group was then transformed into a new mixed feature so that the dimension of dataset was further reduced.

The application of NMFBFS on a clinical dataset of HCC proved the effectiveness of this approach. The optimal clinical features derived from NMFBFS approach contained many well-recognized symptoms of HCC patients. Moreover, this study also provides a general computational framework of a novel feature selection approach to efficiently extract the optimal feature subset from a high-dimensional dataset.

## Supplementary Material

Supplementary information includes two Figures and one Table.
Fig S1: denotes the results of feature ranking with ReliefF.
Fig S2: denotes the results of feature selection with Elastic Net.
Table S1: represents the frequencies of each symptom feature appearing positive over the samples in all the clinical stages.

## Figures and Tables

**Table 1:** The description of original clinical data of HCC patients.

Sex	Phase I (82)		Phase II (195)		Phase III (130)	
	Phase IA	Phase IB	Phase IIA	Phase IIB	Phase IIIA	Phase IIIB
Male	33	27	50	115	95	10
Female	12	10	10	20	16	9

**Table 2:** Eight irrelevant symptoms were screened with threshold 10%. Each of them is rarely positive in each phase.

Symptoms	Phase I		Phase II		Phase III	
	Phase IA	Phase IB	Phase IIA	Phase IIB	Phase IIIA	Phase IIIB
Pale white lip [*V*_1_]	0	5.41%	6.67%	5.19%	4.5%	0
Edema in lower extremities [*V*_16_]	2.22%	8.1%	1.67%	5.19%	3.6%	0
Lack of urine output [*V*_41_]	0	2.7%	0	0	5.41%	0
Emotional depression [*V*_43_]	4.44%	0	5%	8.89%	6.31%	5.26%
Head body trapped heavy [*V*_47_]	0	2.7%	3.33%	2.22%	2.7%	0
Hydrothorax [*V*_51_]	6.67%	2.7%	1.67%	3.7%	2.7%	0
Rapid pulse [*V*_55_]	4.44%	2.7%	1.67%	0.74%	5.41%	5.26%
Uneven pulse [*V*_56_]	4.44%	5.41%	8.33%	3.7%	3.6%	0

**Table 3:** The NMF-derived participation of the symptoms to each corresponding basis component.

Basis components	Number of symptoms	The names of symptoms
Basis 1	16	Varicose veins [*V*_7_]; yellow complexion [*V*_11_]; yellow skin and eye [*V*_13_]; stomach pain [*V*_31_]; dry stool [*V*_38_]; feeling thirsty [*V*_27_]; hot flash [*V*_20_]; doing belly full bilge [*V*_33_]; fullness in stomach [*V*_32_]; block under the rib [*V*_49_]; chills [*V*_18_]; fever [*V*_19_]; spider telangiectasia in liver palm [*V*_15_]; ascites [*V*_50_]; yellow greasiness [*V*_9_]; anorexia [*V*_34_]

Basis 2	17	Nausea [*V*_35_]; pulse slip [*V*_54_]; petechial and ecchymosis tongue [*V*_6_]; white slip [*V*_8_]; chest distress [*V*_28_]; semiliquid stool [*V*_37_]; weak pulse [*V*_53_]; night sweat [*V*_22_]; dirty mouth [*V*_17_]; red tongue [*V*_3_]; thready pulse [*V*_57_]; sticky greasy coating [*V*_10_]; purple tongue [*V*_4_]; stringy pulse [*V*_52_]; pale white lip [*V*_2_]; large and teeth-printed tongue [*V*_5_]; gloomy complexion [*V*_14_]

Basis 3	16	Tinnitus [*V*_24_]; dizziness [*V*_23_]; pain in shoulder and back [*V*_48_]; chest pain [*V*_29_]; distending pain in hypochondrium [*V*_30_]; bitter taste [*V*_26_]; insomnia [*V*_42_]; appearance with stained yellow [*V*_12_]; yellow urine [*V*_40_]; hiccup [*V*_36_]; soreness and weakness of waist and knees [*V*_44_]; dry throat [*V*_25_]; feverishness in palms and soles [*V*_45_]; spontaneous perspiration [*V*_21_]; night urination much [*V*_39_]; physically and mentally fatigued [*V*_46_]

**Table 4:** The mean similarity values about the pairs of redundant symptoms within the same groups.

Basis components	The screened redundant symptoms	Distance-based similarity sim_dist(*w*_*i*_, *w*_*j*_)	Correlation-based similarity sim_corr(*w*_*i*_, *w*_*j*_)
Basis 1	*V*_38_,*V*_27_, *V*_20_	0.9672	1.0
	*V*_19_,*V*_15_	0.9507	1.0

Basis 2	*V*_35_,*V*_54_	0.9685	0.9960
	*V*_6_,*V*_8_,*V*_53_,*V*_37_,*V*_28_	0.9628	1.0

Basis 3	*V*_48_,*V*_29_	0.9686	1.0
	*V*_44_,*V*_45_	0.9520	0.9926

**Table 5:** The NMF-driven potential clinical features of HCC (threshold: 0.95).

Basis components	Original features	Mixed features	Description about mixed features
Basis 1	*V*_7_; *V*_11_; *V*_13_; *V*_31_; *V*_33_; *V*_32_; *V*_49_; *V*_18_; *V*_50_; *V*_9_; *V*_34_	*M*_11_ *M*_12_	Converted from {*V*_38_, *V*_27_, *V*_20_} and {*V*_19_, *V*_15_}, respectively.

Basis 2	*V*_22_; *V*_17_; *V*_3_; *V*_57_; *V*_2_; *V*_10_; *V*_4_; *V*_52_; *V*_5_; *V*_14_	*M*_21_ *M*_22_	Converted from {*V*_35_, *V*_54_} and {*V*_6_, *V*_8_, *V*_53_, *V*_37_, *V*_28_}, respectively.

Basis 3	*V*_24_; *V*_23_; *V*_30_; *V*_26_; *V*_42_; *V*_12_; *V*_40_; *V*_36_; *V*_25_; *V*_21_; *V*_39_; *V*_46_	*M*_31_ *M*_32_	Converted from {*V*_48_, *V*_29_} and {*V*_44_, *V*_45_}, respectively.

Number of features	33	6	Total: 39 features

**Table 6:** Classification accuracy among three feature subsets on the training set (120 representative samples). FS_1_ was obtained by the proposed approach with a given threshold (*θ* = 0.95), in which 33 original symptom features and 6 new mixed features were included. FS_2_ denotes the above-mentioned 33 original symptom features (FS_2_ ⊂ FS_1_). OFS indicates all the 49 symptoms before NMF calculation.

Feature subsets	Dimension	Classification accuracy in LSSVM (%)
FS_1_	39	80.002 ± 9.95
FS_2_	33	77.50 ± 12.36
OFS	49	72.50 ± 11.64

**Table 7:** Classification accuracy of inferred optimal feature subset via NMFBFS, ReliefF, mRMR, and Elastic Net on the training set.

Methods	Feature subset	Dimension	Classification accuracy in LSSVM (%)
**NMFBFS**	**F** **S**_1_	**39**	**80.002 ± 9.95**

ReliefF	FS_RF20_	20	65.00 ± 10.03
	FS_RF40_	40	73.33 ± 15.76

mRMR	FS_MR20_	20	70.83 ± 12.5
	FS_MR40_	40	74.17 ± 9.03

Elastic Net	FS_EN20_	20	70.00 ± 11.56
	FS_EN40_	40	76.67 ± 10.46

**Table 8:** Classification accuracy of inferred optimal feature subset via NMFBFS, ReliefF, mRMR, and Elastic Net on the testing set.

Methods	Feature subset	Dimension	Classification accuracy in LSSVM (%)
**NMFBFS**	**F** **S**_1_	**39**	**79.65 ± 6.48**

ReliefF	FS_RF20_	20	50.71 ± 1.22
	FS_RF40_	40	76.43 ± 8.27

mRMR	FS_MR20_	20	63.79 ± 1.22
	FS_MR40_	40	77.14 ± 9.18

Elastic Net	FS_EN20_	20	67.57 ± 4.09
	FS_EN40_	40	78.38 ± 9.62

**Table 9:** The performance of classification for the inferred optimal feature subsets with different threshold *θ*.

The values of *θ*	Original symptom features	New mixed features	Total number of features	Classification accuracy (%)
*θ* = 0.95	33	6	39	80.002 ± 9.95
*θ* = 0.90	21	9	30	70.83 ± 6.59
*θ* = 0.85	10	8	18	70.00 ± 4.56

## Abbreviations

HCC: Hepatocellular carcinoma
TCM: Traditional Chinese Medicine
NMF: Nonnegative matrix factorization
LSSVM: Least squares support vector machines
KNN: *K*-nearest neighbor.
