# Supplementary material for: NMFBFS: A NMF-Based Feature Selection Method in Identifying Pivotal Clinical Symptoms of Hepatocellular Carcinoma
Source: Comput Math Methods Med. 2015 Oct 12;2015:846942. doi: 10.1155/2015/846942 (PMC4633688; doi:10.1155/2015/846942)
Supplement: Supplementary file 1 — Supplementary information includes two Figures and one Table. Fig S1: denotes the results of feature ranking with ReliefF. Fig S2: denotes the results of feature selection with Elastic Net. Table S1: represents the frequencies of each symptom feature appearing positive over the samples in all the clinical stages. [file 846942.f1.pdf]

NMFBFS: a NMF-based feature selection method in identifying pivotal clinical symptoms of Hepatocellular carcinoma

Zhiwei Ji[1,2,☼], Guanmin Meng[3,☼], Deshuang Huang[1], Xiaoqiang Yue[4,*],Bing Wang[1,5,6,*]

[1]Machine Learning & Systems Biology Lab, School of Electronics and Information Engineering, Tongji University, 4800 Caoan Road, Shanghai 201804, China

[2]School of Information Engineering, Zhejiang A&F University, 88 Huancheng North Road, Linan 311300, China

[3]Department of Clinical Laboratory, Tongde Hospital of Zhejiang Province, 234th Gucui Road, Hangzhou 310012, China

[4]Department of Traditional Chinese Medicine, Changzheng Hospital, Second Military Medical University, 415 Fengyang Road, Shanghai 200003, China

[5]The Advanced Research Institute of Intelligent Sensing Network, Tongji University, 4800 Caoan Road, Shanghai 201804, China

[6]The Key Laboratory of Embedded System and Service Computing, Tongji University, 4800 Caoan Road, Shanghai 201804, China

Supplementary Materials

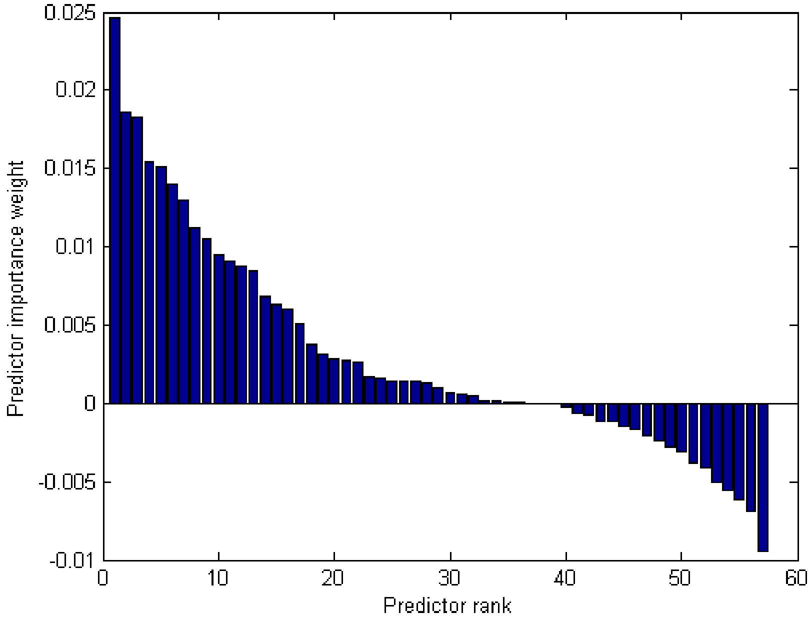

Fig. S1 ReliefF calculated the weight of each feature in original dataset.

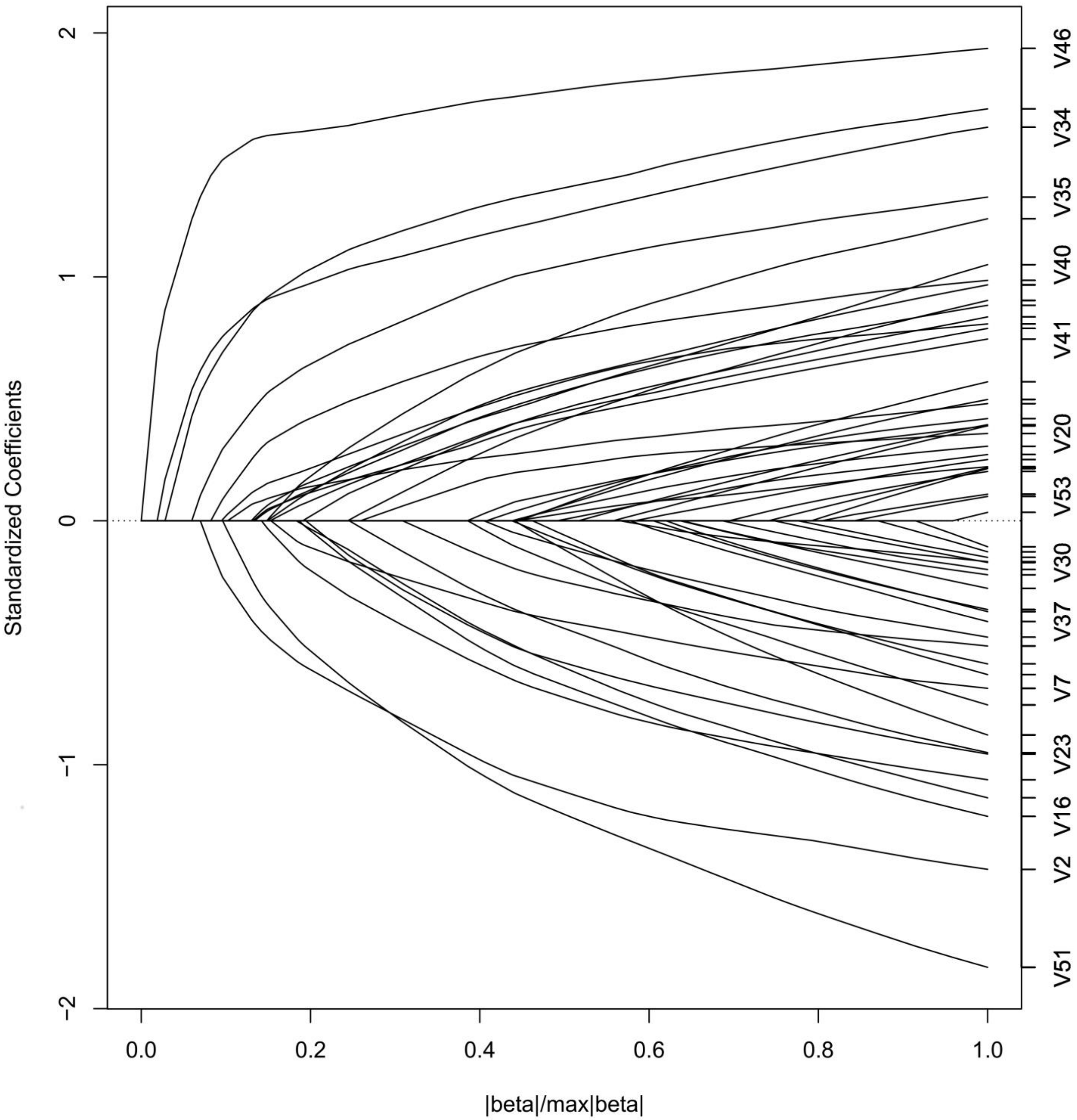

Fig. S2 Elastic net (λ=0.5) solution paths for feature selection.

Table S1. The frequencies of each symptom feature appearing positive over the samples in all the clinical stages

symptoms	Phase I		Phase II		Phase III	
	Phase IA	Phase IB	Phase IIA	Phase IIB	Phase IIIA	Phase IIIB
V1	0.00%	5.41%	6.67%	5.19%	4.50%	0.00%
V2	51.11%	40.54%	45.00%	34.07%	33.33%	26.32%
V3	42.22%	21.62%	33.33%	29.63%	38.74%	47.37%
V4	68.89%	56.76%	60.00%	54.07%	54.05%	52.63%
V5	40.00%	29.73%	53.33%	44.44%	45.05%	36.84%
V6	22.22%	10.81%	11.67%	14.07%	19.82%	21.05%
V7	37.78%	29.73%	31.67%	17.78%	25.23%	21.05%
V8	8.89%	8.11%	21.67%	13.33%	16.22%	5.26%
V9	48.89%	43.24%	36.67%	38.52%	42.34%	31.58%
V10	64.44%	54.05%	63.33%	60.00%	61.26%	47.37%
V11	15.56%	8.11%	10.00%	17.04%	18.92%	5.26%
V12	26.67%	27.03%	31.67%	29.63%	22.52%	42.11%
V13	6.67%	8.11%	10.00%	10.37%	7.21%	0.00%
V14	77.78%	75.68%	60.00%	60.00%	53.15%	57.89%
V15	33.33%	21.62%	41.67%	28.89%	32.43%	5.26%
V16	2.22%	8.11%	1.67%	5.19%	3.60%	0.00%
V17	11.11%	10.81%	8.33%	12.59%	12.61%	26.32%
V18	13.33%	27.03%	21.67%	29.63%	18.02%	36.84%
V19	13.33%	16.22%	20.00%	17.04%	21.62%	26.32%
V20	11.11%	2.70%	8.33%	8.89%	12.61%	21.05%
V21	20.00%	16.22%	15.00%	13.33%	18.92%	15.79%
V22	22.22%	29.73%	25.00%	20.00%	23.42%	31.58%
V23	17.78%	35.14%	20.00%	25.19%	22.52%	10.53%
V24	22.22%	24.32%	13.33%	14.07%	16.22%	10.53%
V25	37.78%	51.35%	33.33%	46.67%	62.16%	63.16%
V26	28.89%	35.14%	31.67%	34.07%	45.95%	36.84%
V27	11.11%	8.11%	8.33%	8.89%	15.32%	5.26%
V28	11.11%	8.11%	11.67%	11.11%	18.92%	21.05%
V29	0.00%	0.00%	6.67%	1.48%	2.70%	10.53%
V30	46.67%	59.46%	56.67%	45.19%	57.66%	57.89%
V31	15.56%	10.81%	15.00%	11.11%	9.91%	10.53%
V32	24.44%	32.43%	30.00%	29.63%	32.43%	47.37%
V33	8.89%	24.32%	13.33%	18.52%	21.62%	26.32%
V34	17.78%	21.62%	21.67%	27.41%	39.64%	21.05%
V35	13.33%	13.51%	13.33%	15.56%	26.13%	21.05%
V36	22.22%	32.43%	30.00%	32.59%	34.23%	21.05%

V37	28.89%	27.03%	20.00%	17.04%	24.32%	26.32%
V38	11.11%	5.41%	6.67%	10.37%	11.71%	21.05%
V39	33.33%	27.03%	31.67%	21.48%	28.83%	31.58%
V40	22.22%	32.43%	23.33%	39.26%	34.23%	52.63%
V41	0.00%	2.70%	0.00%	0.00%	5.41%	0.00%
V42	24.44%	29.73%	21.67%	25.19%	36.94%	42.11%
V43	4.44%	0.00%	5.00%	8.89%	6.31%	5.26%
V44	33.33%	43.24%	38.33%	29.63%	32.43%	31.58%
V45	13.33%	18.92%	16.67%	14.07%	22.52%	15.79%
V46	40.00%	51.35%	40.00%	59.26%	65.77%	57.89%
V47	0.00%	2.70%	3.33%	2.22%	2.70%	0.00%
V48	0.00%	2.70%	1.67%	5.19%	2.70%	10.53%
V49	4.44%	18.92%	13.33%	16.30%	28.83%	10.53%
V50	13.33%	16.22%	15.00%	26.67%	27.93%	42.11%
V51	6.67%	2.70%	1.67%	3.70%	2.70%	0.00%
V52	73.33%	56.76%	61.67%	58.52%	63.06%	52.63%
V53	24.44%	16.22%	18.33%	21.48%	23.42%	15.79%
V54	22.22%	21.62%	28.33%	16.30%	18.92%	10.53%
V55	4.44%	2.70%	1.67%	0.74%	5.41%	5.26%
V56	4.44%	5.41%	8.33%	3.70%	3.60%	0.00%
V57	35.56%	40.54%	45.00%	37.04%	41.44%	42.11%
